# Utilization of SYNTAX Score II for Predictive Clinical Outcomes in Patients with Coronary Artery Disease and Chronic Renal Insufficiency Following Percutaneous Coronary Intervention

**DOI:** 10.31083/j.rcm2510371

**Published:** 2024-10-22

**Authors:** Liqiu Yan, Dong Han, Yabin Wang, Sulei Li, Wei Yan, Nan Guo, Ying Mao, Qian Yang, Mengyao Li, Yumeng Lei, Shuaiyong Zhang, Feng Cao

**Affiliations:** ^1^Department of Cardiology, the Affiliated Dongguan Songshan Lake Central Hospital, Guangdong Medical University, 523326 Dongguan, Guangdong, China; ^2^The Second Medical Center & National Clinical Research Center for Geriatric Diseases, Chinese PLA General Hospital, 100853 Beijing, China; ^3^Department of Cardiology, Cangzhou Central Hospital, Hebei Medical University, 061001 Cangzhou, Hebei, China

**Keywords:** percutaneous coronary intervention, coronary artery disease, SYNTAX score, SYNTAX score II, chronic renal insufficiency

## Abstract

**Background:**

The SYNTAX score II (SS II) has earned widespread recognition for use on individuals with coronary artery disease (CAD) due to its reliable predictions of 4-year all-cause mortality (ACM). This research focuses on substantiating the prognostic significance of using the SS II for patients experiencing concurrent chronic renal insufficiency (CRI) and CAD who have undergone percutaneous coronary intervention (PCI).

**Methods:**

This study retrospectively examined 2468 patients with concurrent CAD and CRI who underwent PCI. Based on their SS II, these participants were sorted into low-, medium-, and high-risk groups and monitored over a median of three years. The evaluation of the predictive precision of different SYNTAX scores for clinical outcomes in patients with CRI after PCI involved using time-dependent receiver operating characteristic (ROC) curves. These included the standard SS (SS), SS II, clinical SS (CSS), and residual SS (rSS). The primary outcomes were ACM and cardiac mortality (CM), while the secondary outcomes covered major adverse cardiovascular and cerebrovascular events (MACCEs), stroke, unplanned revascularization, and myocardial infarction (MI).

**Results:**

Higher 5-year cumulative incidences of MACCEs, MI, CM, and ACM were observed significantly in patients in the high SS II category relative to those in the low and medium SS II categories. Multivariable Cox regression analysis confirmed that the SS II independently predicts ACM, CM, MI, and MACCEs as a prognostic marker. Additionally, the analysis of the time-dependent ROC curve demonstrated that the areas under the curve (AUC) for predicting CM and ACM were 0.772 and 0.767, respectively, which are superior to those of other SYNTAX scores (*p* < 0.05).

**Conclusions:**

As an independent predictor, the SS II is notable for its ability to forecast long-term adverse outcomes, including MACCEs, CM, ACM, and MI. For patients with coexisting CAD and CRI undergoing PCI, it provides significantly improved prognostic accuracy for 5-year ACM and CM compared to other SYNTAX scores.

## 1. Introduction

In individuals experiencing coronary artery disease (CAD), chronic renal insufficiency (CRI) is a recognized independent risk factor for mortality and cardiovascular events [1]. Patients with CRI are more susceptible to major adverse cardiovascular and cerebrovascular events (MACCEs), which include sudden cardiac mortality (CM), stroke, congestive heart failure, and myocardial infarction (MI) [2]. Interestingly, compared to individuals progressing to end-stage renal disease (ESRD) and needing renal replacement therapy, those with CRI experience a higher incidence of MACCEs [3]. Within the framework of percutaneous coronary intervention (PCI), CRI correlates with reduced rates of procedural success and a heightened risk of complications, including stent thrombosis and restenosis [4].

In patients experiencing complex coronary artery disease (CCAD), the SYNTAX score (SS), a widely validated instrument to guide revascularization strategies, aids clinicians in tailoring treatment more precisely—its prognostic utility shines in patients undergoing PCI [5, 6, 7, 8]. Recent developments have led to the introduction of the residual SYNTAX score (rSS) [9, 10], clinical SYNTAX score (CSS) [11], and SYNTAX score II (SS II) [12]. SS II was created by integrating anatomical and clinical variables to enhance long-term prognostic predictions. These variables include six clinical variables (left ventricular ejection fraction (LVEF), chronic obstructive pulmonary disease (COPD), peripheral vascular disease, gender, creatinine clearance rate, and age) and two anatomical factors (SS and unprotected left main coronary artery (ULMCA)). Its effectiveness has been demonstrated through external validation in the DELTA registry and internal validation in the SYNTAX trial, showing superior 4-year all-cause mortality (ACM) predictions for patients with CCAD [13, 14, 15]. Despite the proven predictive power of rSS and CSS in CRI patients post-PCI [16, 17], research on the prognostic efficacy of SS II in this particular cohort is lacking. Therefore, evaluating the prognostic implications of SS II in patients experiencing concurrent CAD and CRI undergoing PCI is the main goal of this research.

## 2. Materials and Methods

### 2.1 Study Population

A prior publication provides a detailed exposition of the methodology employed to evaluate risk in patients with CRI undergoing PCI [16]. From January 2014 to September 2017, our single-center retrospective study included 2468 patients who, with a creatinine clearance (CrCl) rate below 90 mL/min per 1.73 m^2^, underwent PCI. Patients were stratified into three categories according to their SS II, which were optimized for predicting ACM using X-tile software [18]. The group classifications were as follows: low SS II group, with SS II ≤29.5 (n = 1,456); median SS II group, with SS II >29.5 and <37.3 (n = 764); high SS II group, with SS II ≥37.3 (n = 248).

Information regarding demographic, clinical, angiographic, and laboratory variables was gathered from the electronic medical records (EMRs) system. Further, CrCl was determined by employing the simplified Modification of Diet in Renal Disease (MDRD) equation. Two senior cardiologists, blind to patient outcomes, assessed coronary angiograms and assigned a SS to coronary lesions exhibiting a stenosis of 50% or more in vessels with a diameter of at least 1.5 mm. When disagreements arose, a third cardiologist was consulted to provide a consensus.

Furthermore, the rSS was determined based on the remaining obstructive coronary disease post-PCI. The CSS for each patient was calculated as follows: CSS = [SS] × [age, creatinine, and ejection fraction (ACEF)_CrCl_ score]. In the ACEF_CrCl_ score calculation, age and ejection fraction were adjusted by an additional point for every 10 mL/min decrease in CrCl below 60 mL/min/1.73 m^2^. The SS II for both coronary artery bypass grafting (CABG) and PCI was derived from six clinical variables (peripheral vascular disease, COPD, sex, LVEF, age, and CrCl) and two anatomical variables (ULMCAD and SS), as previously described [12].

The study adhered to the ethical guidelines of the Declaration of Helsinki, and ethical clearance was granted by the Ethics Committee of Cangzhou Central Hospital, which is affiliated with Hebei Medical University. Written informed consent was secured from all participants.

### 2.2 Study Endpoints

Our study primarily investigated two endpoints: ACM and CM. Additionally, we considered secondary endpoints, including MI, unplanned revascularization, stroke, and MACCEs. The criteria for identifying MI were previously outlined [19]. Any revascularization procedure, whether PCI or CABG, prompted by cardiac events or symptoms of ischemia was categorized as unplanned revascularization. Two cardiologists independently determined the endpoint.

### 2.3 Statistical Analysis

Based on the statistical distribution, continuous variables are reported as the mean ± standard deviation or median with interquartile range (IQR). Normal distribution was assessed using one-way analysis of variance (ANOVA); non-normally distributed variables were evaluated utilizing the Kruskal–Wallis test. Categorical variables are expressed as counts (percentages), and comparisons were made using either the Chi-square test or Fisher’s exact tests, as appropriate.

The Kaplan–Meier method was employed to analyze survival outcomes, estimating 5-year cumulative event rates using the log-rank test for statistical comparisons. Multivariable Cox regression analysis, employing the enter method, was utilized to determine the relationship between the SS II and clinical outcomes while accounting for other variables known to affect long-term mortality. Proportional hazard assumptions were verified using Log[-logS(t)] plots.

Furthermore, we assessed the prognostic accuracy of four risk scores (SS, rSS, CSS, and SS II) using time-dependent receiver operating characteristic (ROC) curves at 60 months. Utilizing the DeLong method, we compared values of the area under the curve (AUC). To assess the ability of SS II to reclassify risks for ACM and CM compared to anatomical SS, integrated discrimination improvement (IDI) and net reclassification improvement (NRI) indices were calculated [20].

Statistical significance was determined by a two-sided *p*-value of less than 0.05. Analyses were performed through SPSS version 24.0 (IBM-SPSS Statistics, Chicago, IL, USA) and R package version 3.6.0 (R Foundation for Statistical Computing, Vienna, Austria), utilizing specific packages such as survival, survIDINRI, timeROC, survminer, and nricens for various analyses.

## 3. Results

### 3.1 Baseline Clinical and Procedural Characteristics

Stratification of patients’ baseline clinical and procedural characteristics following the SS II for PCI is meticulously outlined in Table 1 and **Supplementary Table 1**. Higher SS II were associated with older age, female gender, and increased prevalence of comorbidities, such as diabetes, MI, stroke, and COPD (all *p* < 0.05). Additionally, patients showing higher SS II exhibited lower estimated glomerular filtration rate (eGFR), LVEF, and hemoglobin levels but higher levels of low-density lipoprotein, high-density lipoprotein, triglycerides, total cholesterol, and fasting glucose (*p* < 0.05 for all).

**Table 1.** **Baseline clinical characteristics**.

		Low SS II group	Median SS II group	High SS II group	*p*-value
		(n = 1456)	(n = 764)	(n = 248)	
Age, years		62.0 (57.0–68.0)	69.0 (65.0–73.0)	72.5 (68.0–77.0)	<0.001
Male		1159 (79.6%)	230 (30.1%)	63 (25.4%)	<0.001
BMI, kg/m^2^		26.1 (23.9–28.0)	25.8 (23.4–28.4)	26.2 (22.6–27.6)	0.273
Diabetes		279 (19.2%)	198 (25.9%)	89 (35.9%)	<0.001
Hypertension		953 (65.5%)	527 (69.0%)	178 (71.8%)	0.065
Dyslipidemia		576 (39.6%)	298 (39.0%)	99 (39.9%)	0.955
Current smoker		179 (12.3%)	76 (10.0%)	20 (8.1%)	0.066
Medical history					
	Prior MI	112 (7.7%)	63 (8.3%)	33 (13.3%)	0.013
	Previous PCI	204 (14.0%)	89 (11.7%)	29 (11.7%)	0.234
	Previous stroke	139 (9.6%)	98 (12.8%)	32 (12.9%)	0.035
	COPD, n (%)	11 (0.8%)	14 (1.8%)	15 (6.1%)	<0.001
Clinical presentation					<0.001
	Stable angina	629 (43.2%)	315 (41.2%)	66 (26.6%)	
	Unstable angina	187 (12.8%)	105 (13.7%)	23 (9.3%)	
	NSTEMI	233 (16.0%)	134 (17.5%)	53 (21.4%)	
	STEMI	407 (28.0%)	210 (27.5%)	106 (42.7%)	
Renal function					<0.001
	eGFR, mL/min	82.5 (76.1–86.6)	74.2 (66.4–80.9)	54.8 (43.9–66.9)	<0.001
	60 ≤ eGFR < 90 mL/min	1412 (97.0%)	669 (87.6%)	92 (37.1%)	
	30 ≤ eGFR < 60 mL/min	44 (3.0%)	94 (12.3%)	133 (53.6%)	
	eGFR <30 mL/min	0 (0.0%)	1 (1.3%)	23 (9.3%)	
	LVEF, %	63.0 (58.0–66.0)	61.0 (55.0–65.0)	53.0 (40.0–63.0)	<0.001
	LVEDD (mm)	48.0 (45.0–51.0)	47.0 (44.0–50.0)	48.0 (44.5–55.0)	<0.001
Baseline laboratory					
	Hemoglobin (mg/dL)	13.7 (12.8–14.7)	12.6 (11.7–13.6)	11.7 (10.7–12.9)	<0.001
	Creatinine (mg/dL)	1.0 (0.9–1.0)	0.9 (0.8–1.0)	1.1 (0.9–1.4)	<0.001
	Fasting glucose (mg/dL)	113.4 (95.4–147.6)	120.6 (98.6–164.2)	141.7 (108.0–187.0)	<0.001
	TG (mg/dL)	132.9 (95.9–192.2)	134.6 (96.5–184.9)	136.4 (103.6–187.8)	0.854
	TC (mg/dL)	166.3 (143.1–193.4)	170.7 (147.3–202.0)	177.9 (151.0–208.0)	<0.001
	HDL (mg/dL)	35.2 (30.2–41.0)	36.4 (31.3–42.9)	36.7 (30.2–43.7)	0.003
	LDL (mg/dL)	95.9 (77.8–117.9)	98.2 (80.8–120.3)	101.1 (84.8–126.1)	0.030

Values are presented as the mean ± SD, median (interquartile range), or n (%). BMI, body mass index; MI, myocardial infarction; PCI, percutaneous coronary intervention; COPD, chronic obstructive pulmonary disease; NSTEMI, non-ST-segment elevation myocardial infarction; SS II, SYNTAX score II; STEMI, ST-segment elevation myocardial infarction; eGFR, estimated glomerular filtration rate; LVEF, left ventricular ejection fraction; LVEDD, left ventricular end-diastolic diameter; TG, triglyceride; HDL, high-density lipoprotein; LDL, low-density lipoprotein.

The median scores for the SS, rSS, CSS, SS II for PCI, and SS II for CABG were 13.0 (8.0–19.0), 5.0 (1.0–10.0), 16.3 (10.2–25.3), 28.0 (23.8–32.6), and 24.0 (19.1–29.0), respectively. Most patients (68.2%) had a higher SS II for PCI than CABG, while 31.2% had a lower SS II for PCI, and 0.6% had equivalent scores. Patients with high SS II presented with more anatomically complicated diseases, as indicated by the higher SS and rSS (*p* < 0.001).

### 3.2 Follow-up and Clinical Outcomes

**Fig. 1.**
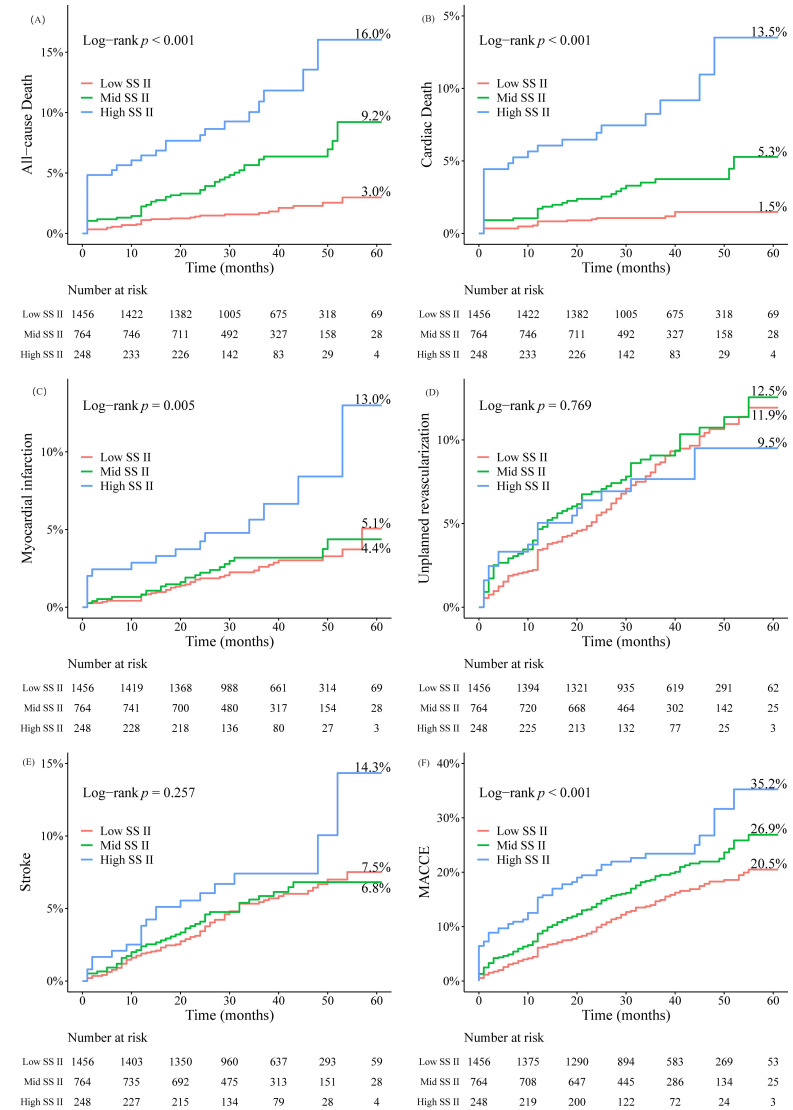
**Kaplan–Meier curves stratified by the SS II over five years**. (A) All-cause death. (B) Cardiac death. (C) Myocardial infarction. (D) Unplanned revascularization. (E) Stroke. (F) MACCE. MACCE, major adverse cardiovascular and cerebrovascular event; SS II, SYNTAX score II.

**Fig. 2.**
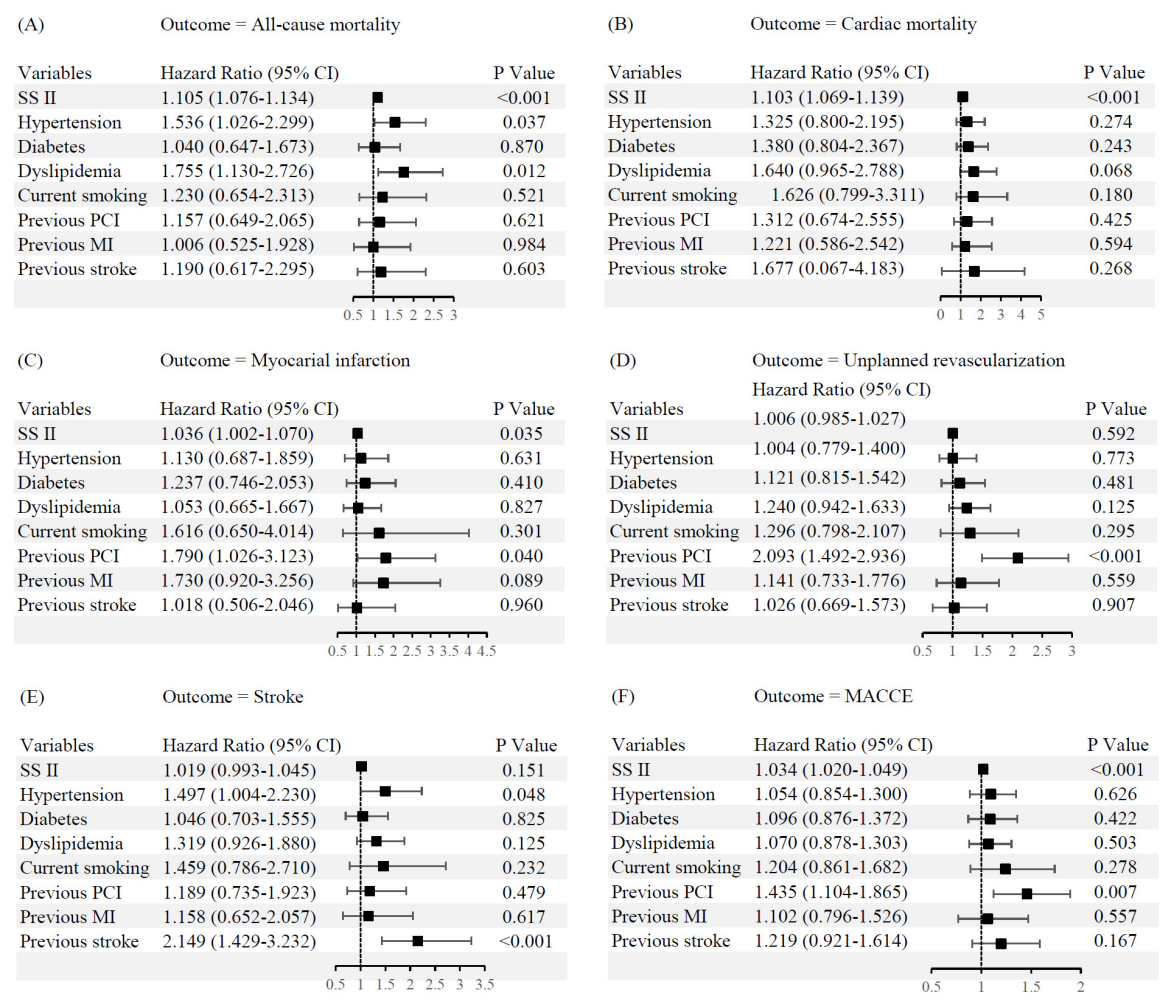
**Independent predictors of long-term clinical outcomes**. (A) All-cause mortality. (B) Cardiac mortality. (C) Myocardial infarction. (D) Unplanned revascularization. (E) Stroke. (F) MACCE. CI, confidence interval; MACCE, major adverse cardiovascular and cerebrovascular event; SS II, SYNTAX score II; MI, myocardial infarction; PCI, percutaneous coronary intervention.

Of the participants, 98.3% completed the median 3-year follow-up from 1.5 to 5.0 years. Outcomes were stratified based on SS II, as shown in Fig. 1 and **Supplementary Table 2**. The high SS II group exhibited the highest 5-year cumulative event rates for ACM (3.0% vs. 9.2% vs. 16.0%, *p* < 0.001), CM (1.5% vs. 5.3% vs. 13.5%, *p* < 0.001), MI (5.1% vs. 4.4 vs. 13.0%, *p* = 0.005), and MACCEs (20.5% vs. 26.9% vs. 35.2%, *p* < 0.001). Multivariate Cox regression identified SS II as an independent predictor for MACCEs (hazard ratio (HR): 1.034, 95% confidence interval (CI): 1.020–1.049; *p* < 0.001), MI (HR: 1.036, 95% CI: 1.002–1.070; *p* = 0.035), CM (HR: 1.103, 95% CI: 1.069–1.139; *p* < 0.001), ACM (HR: 1.105, 95% CI: 1.076 to 1.134; *p* < 0.001) (Fig. 2).

### 3.3 Predictive Efficacy of SS II Compared to Other Risk Scores 

**Fig. 3.**
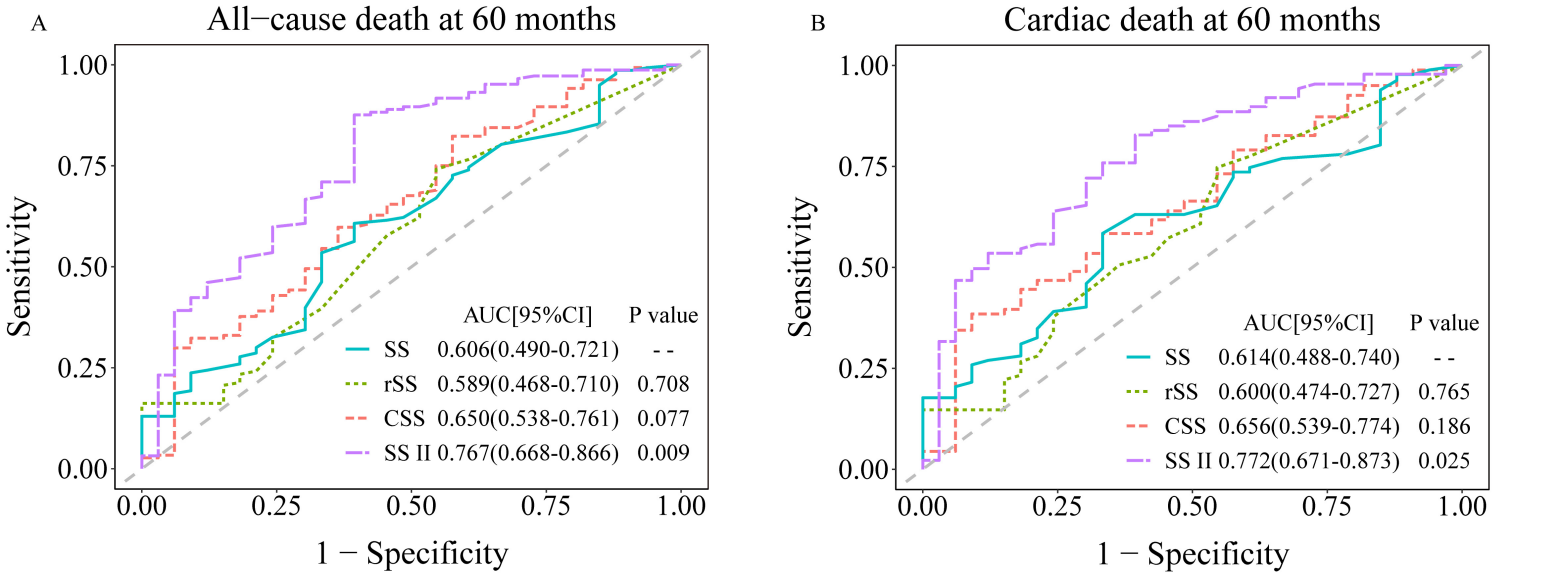
**Time-dependent ROC curve analyses at 36 months comparing SS with rSS, CSS, and SS II for the predictability of long-term all-cause death (A) and cardiac death (B)**. AUC, area under the curve; CI, confidence interval; CSS, clinical SYNTAX score; rSS, residual SYNTAX score; SS, SYNTAX score; SS II, SYNTAX score II; ROC, receiver operating characteristic.

Time-dependent ROC curves (at 60 months) analyses demonstrated that the AUCs for SS, rSS, CSS, and SS II for ACM were 0.606, 0.589, 0.650, and 0.767, respectively, and were 0.614, 0.600, 0.656, and 0.772, respectively, for CM. The AUCs for SS II, combining clinical variables with anatomic SS, showed a significant increase (*p* < 0.05 for all). Meanwhile, the AUCs for CSS exhibited an increasing trend compared with SS, although not statistically significant (Fig. 3). Furthermore, over a median follow-up period of 3 years, CSS and SS II demonstrated superior predictive capabilities for ACM and CM compared to SS, significantly improving risk stratification and discrimination. The NRI of CSS and SS II over SS was 15.8% and 44.2%, respectively, for ACM and 9.2% and 25.3%, respectively, for CM. The respective IDI indices of CSS and SS II over SS for ACM were 0.011 (*p* = 0.020) and 0.027 (*p* < 0.001), and for CM, were 0.012 (*p* = 0.010) and 0.020 (*p* < 0.001) (Table 2).

**Table 2.** **Statistics for model improvement of rSS, CSS, SS II over SS for all-cause and cardiac mortality**.

		Model performance		Risk reclassification	Discrimination	
		AUC (95% CI)	*p*-value	NRI (95% CI)	IDI (95% CI)	*p*-value
All-cause mortality						
	SS	0.612 (0.546–0.677)	–	–	–	–
	rSS	0.599 (0.534–0.665)	0.652	–0.024 (–0.125–0.107)	0.000 (–0.004–0.009)	0.896
	CSS	0.666 (0.603–0.728)	0.001	0.158 (–0.083–0.384)	0.011 (0.000–0.003)	0.020
	SS II	0.728 (0.676–0.779)	<0.001	0.442 (0.234–0.598)	0.027 (0.012–0.060)	<0.001
Cardiac mortality						
	SS	0.664 (0.566–0.722)	–	–	–	–
	rSS	0.606 (0.526–0.686)	0.251	0.002 (–0.221–0.145)	–0.001 (–0.007–0.012)	0.806
	CSS	0.698 (0.621–0.774)	0.005	0.092 (–0.084–0.271)	0.012 (0.002–0.036)	0.010
	SS II	0.732 (0.669–0.796)	0.025	0.253 (–0.051–0.406)	0.020 (0.006–0.053)	<0.001

AUC, area under the curve; CSS, clinical SYNTAX score; IDI, integrated discrimination improvement; NRI, net reclassification improvement; rSS, residual SYNTAX score; SS, SYNTAX score; SS II, SYNTAX score II.

## 4. Discussion

Our research is pioneering in validating the SS II in forecasting long-term clinical outcomes for patients suffering from CRI undergoing PCI. Notably, within this patient group, the SS II outperforms SS in predictive performance for ACM and CM.

Prior research has underscored the enhanced predictive power of risk scores that combine anatomical SS with clinical variables, compared to SS alone. Correspondingly, the clinical SS, integrating SS, and the modified ACEF score have demonstrated superior accuracy in predicting 5-year mortality and MACCEs in CCAD patients who have received PCI [11]. Additionally, the prognostic efficacy of this combined score (CSS) was externally validated in the SIRTAX trial [21]. Interestingly, while the CSS exhibited a trend toward improved prediction of 5-year ACM and CM in our study, this was not statistically significant. Moreover, the particular attributes of our study population, focusing on patients with CRI, could potentially account for this observation.

For patients suffering from multivessel disease (MVD) and/or ULMCAD, the SS II emerged from the SYNTAX trial, primarily aimed at refining risk assessment and aiding in choosing the most suitable revascularization strategy [12]. Numerous investigations have underscored the efficacy of the SS II in prognosticating outcomes across both PCI and CABG cohorts. Research by Campos *et al*. [13] showcased the superior predictive accuracy of the SS II over the anatomical SS, with respective C-index values of 0.75 and 0.70, alongside enhanced risk stratification (NRI = 0.5, *p* < 0.01) across both PCI and CABG groups. Furthermore, Xu *et al*. [22] validated the enduring prognostic prowess of the SS II in a large real-world cohort of consecutive patients undergoing PCI for ULMCAD, revealing superior predictive performance (C-index: 0.694 vs. 0.591, *p* < 0.001), improved risk reclassification (NRI = 0.25, *p* = 0.002), and heightened discrimination (IDI = 0.015, *p* = 0.003) for long-term mortality compared to strictly anatomical SS. Additionally, Sotomi *et al*. [23] corroborated the ability of the SS II to predict individual long-term mortality following PCI or CABG in a sizable pooled population, demonstrating good calibration and moderate discrimination ability (C-index = 0.685) in patients with MVD and/or ULMCAD. The predictive power of the SS II has been validated across diverse patient populations, including those with three-vessel and/or ULMCAD PCI [24, 25], one- or two-vessel disease [26], acute coronary syndrome [25, 27], left main PCI using second-generation drug-eluting stents (DES) [28, 29], ST-segment elevated myocardial infarction (STEMI) complicated with cardiogenic shock undergoing primary PCI [30], veterans with left main and/or three-vessel disease [31], and octogenarian undergoing PCI [32]. Recent investigations have even suggested the potential of using the SS II in predicting outcomes such as for the “no-reflow phenomenon”, contrast-induced nephropathy (CIN), and hemodialysis in patients with STEMI following primary PCI [33, 34]. Despite these extensive validations, there remains a shortage of literature concerning the applicability of the SS II in patients with CRI undergoing PCI, thereby underscoring the novelty and significance of our study.

CRI has emerged as a potent and independent predictor for a spectrum of adverse cardiovascular outcomes, encompassing mortality, non-fatal MI, and bleeding complications. Despite advancements such as DES and contemporary antithrombotic therapies, these risks retain their significance [35]. Furthermore, the heightened propensity for developing CIN and encountering procedural complications further compounds the prognosis for these individuals, rendering them a particularly vulnerable cohort.

The CSS and SS II are superior predictors of adverse cardiovascular events post-PCI compared to the rSS for several reasons. Firstly, the CSS and SS II encompass a broader spectrum of patient characteristics and disease severity, including comorbidities, anatomical complexity, and procedural outcomes, providing a more comprehensive cardiovascular risk assessment. Secondly, the CSS and SS II incorporate both clinical and anatomical variables, reflecting the intricate interplay between patient factors and coronary artery disease burden. Additionally, the CSS and SS II may capture the impact of post-PCI management strategies and long-term follow-up care, influencing outcomes beyond residual lesion severity alone. Therefore, while the residual burden of coronary artery disease remains important, clinical factors offer a more holistic approach to risk assessment post-PCI, enhancing predictive accuracy and guiding therapeutic decision-making. In a large, real-world cohort, our research definitively demonstrated the superior predictive capability of the SS II for ACM and CM in patients with CRI undergoing PCI, where the AUC for SS II was notably higher for predicting CM (0.772 vs. 0.614, *p* = 0.025) and ACM (0.767 vs. 0.606, *p* < 0.001) compared to the baseline anatomical SS. Moreover, the SS II displayed markedly better risk reclassification and discrimination capabilities: it reclassified 44.2% of patients for ACM and 25.3% for CM compared to the anatomical SS. Concurrently, our findings showed that the CSS, which integrates anatomical and clinical variables, demonstrated an increasing yet non-statistically significant trend in its ability to predict 5-year ACM and CM compared with the SS.

Similar to previous studies [22, 24], the present study also found that the rSS increased with each SS II group. This finding shows the capability of the SS II to pinpoint patients who are more likely to encounter suboptimal revascularization and adverse events. Moreover, similar to prior studies [12, 24, 29], diabetes status was not determined to predict long-term mortality in the multivariate analysis. A recently conducted patient-level pooled analysis from the BEST, SYNTAX, and PRECOMBAT trials, which focused on individuals with MVD, revealed that irrespective of coronary anatomy and other influences, diabetes did not independently affect mortality rates [36]. Interestingly, we have found that previous PCI is the strongest independent predictor for unplanned revascularization, which is consistent with the recent study by Yudi MB *et al*. [37], concluding that previous PCI independently forecasts recurrent acute coronary syndrome or unplanned revascularization hospitalization. Additionally, Arnold *et al*. [38] reported a 4.1% occurrence rate of unplanned revascularization after PCI in 3283 patients with myocardial infarction. However, these models do not consider the physiological impact of the lesions; thus, they may overestimate the anatomical severity of the disease. The importance of functional assessment has been confirmed in numerous studies. Nam *et al*. [39] combined the SS with fractional flow reserve (FFR), introducing the concept of a functional SS. They demonstrated that the combined anatomical and physiological scoring system offers superior discriminative ability in assessing the risk of adverse events. Scala *et al*. [40] recently integrated the SYNTAX score II 2020 with FFR assessment, confirming that integrating functional, anatomical, and clinical data enhances risk prediction and treatment guidance.

## 5. Strengths and Limitations

Patients experiencing CRI and undergoing PCI are the focus of the study, which marks the initial validation of the SS II for predicting long-term clinical results. Nonetheless, several limitations merit acknowledgment. First, the potential bias inherent to the study’s non-randomized design cannot be disregarded. This bias may impact the generalizability and robustness of the findings. Second, the absence of cases involving CABG limits the ability to definitively compare the relative merits of PCI versus CABG using the SS II. Lastly, the invasive characteristics and costs associated with this procedure prevented the integration of fractional flow reserve measurements for evaluating target vessel ischemia in the study.

## 6. Conclusions

This research conclusively shows that for patients with CAD and CRI undergoing PCI, the SS II can independently predict long-term adverse clinical outcomes. Demonstrating superior predictive power over the purely anatomical SS model, the SS II offered enhanced accuracy and improved risk stratification. These findings support the potential of using the SS II model in forecasting long-term prognoses for PCI patients with comorbid CAD and CRI.

## Availability of Data and Materials

The authors are dedicated to furnishing the unprocessed data that underpins the conclusions presented in this article.

## Supplementary Material

Supplementary material associated with this article can be found, in the online version, at https://doi.org/10.31083/j.rcm2510371.
