# Supplementary material for: Utilization of SYNTAX Score II for Predictive Clinical Outcomes in Patients with Coronary Artery Disease and Chronic Renal Insufficiency Following Percutaneous Coronary Intervention
Source: Rev Cardiovasc Med. 2024 Oct 22;25(10):371. doi: 10.31083/j.rcm2510371 (PMC11522769; doi:10.31083/j.rcm2510371)
Supplement: Supplementary file 1 [file 2153-8174-25-10-371-s1.docx]

Supplemental Table 1. Angiographic and Procedural Characteristics

|  | Low SS II group  (n=1456) | Median SS II group  (n=764) | High SS II group  (n=248) | P Value |
| --- | --- | --- | --- | --- |
| No. of diseased vessels |  |  |  | <0.001 |
| 1 | 401 (27.5%) | 106 (13.9%) | 21 (8.5%) |  |
| 2 | 519 (35.7%) | 261 (34.2%) | 63 (25.4%) |  |
| 3 | 536 (36.8%) | 397 (52.0%) | 164 (66.1%) |  |
| Location of diseased vessels |  |  |  |  |
| LM | 72 (5.0%) | 54 (7.1%) | 33 (13.3%) | <0.001 |
| LAD | 451 (78.7%) | 261 (91.6%) | 75 (92.6%) | <0.001 |
| LCX | 332 (57.9%) | 212 (74.4%) | 62 (76.5%) | <0.001 |
| RCA | 388 (67.5%) | 210 (73.7%) | 70 (86.4%) | 0.001 |
| No. of target vessels |  |  |  | 0.180 |
| 1 | 1111 (76.3%) | 548 (71.7%) | 185 (74.6%) |  |
| 2 | 301 (20.7%) | 192 (25.1%) | 57 (23.0%) |  |
| 3 | 44 (3.0%) | 24 (3.1%) | 6 (2.4%) |  |
| Location of target vessels |  |  |  |  |
| LM | 41 (2.8%) | 22 (2.9%) | 8 (3.2%) | 0.938 |
| LAD | 762 (52.3%) | 434 (56.8%) | 134 (54.0%) | 0.133 |
| LCX | 428 (29.4%) | 209 (27.4%) | 61 (24.6%) | 0.238 |
| RCA | 601 (41.3%) | 334 (43.7%) | 112 (45.2%) | 0.356 |
| Procedural characteristics |  |  |  |  |
| Stent per patient | 1.0 (1.0-2.0) | 2.0 (1.0-2.0) | 2.0 (1.0-2.0) | <0.001 |
| Total length of stent, mm | 36.0 (24.0-60.0) | 42.0 (28.0-66.0) | 39.5 (27.3-65.0) | 0.002 |
| Stent length >100 mm | 88 (6.1%) | 57 (7.5%) | 22 (8.9%) | 0.174 |
| Mean stent diameter, mm | 3.0 (2.8-3.3) | 2.9 (2.7-3.0) | 2.8 (2.5-3.0) | <0.001 |
| Minimum stent diameter, mm | 3.0 (2.5-3.0) | 2.8 (2.5-3.0) | 2.8 (2.5-3.0) | <0.001 |
| Maximum stent diameter, mm | 3.0 (2.8-3.5) | 3.0 (2.8-3.5) | 3.0 (2.8-3.5) | <0.001 |
| SYNTAX score | 11.0 (7.0-16.5) | 16.0 (11.0-21.0) | 19.8 (14.4-26.0) | <0.001 |
| SYNTAX score |  |  |  | <0.001 |
| Low (<22) | 1323 (90.9%) | 588 (76.0%) | 147 (59.3%) |  |
| Median (22-32) | 121 (8.3%) | 153 (20.0%) | 76 (30.7%) |  |
| High (>32) | 12 (0.8%) | 23 (3.0%) | 25 (10.1%) |  |
| Residual SS | 3.0 (0.0-8.0) | 7.0 (2.0-11.0) | 8.3 (4.0-15.6) | <0.001 |
| Clinical SS | 13.0 (8.2-18.6) | 20.7 (14.4-29.3) | 46.7 (30.0-67.9) | <0.001 |
| SS II for CABG | 22.3 (20.0-23.8) | 28.0 (26.8-29.5) | 34.7 (32.6-38.0) | <0.001 |
| SS II for PCI | 24.6 (21.8-27.1) | 32.5 (30.9-34.4) | 40.2 (38.5-43.2) | <0.001 |

Value are mean±SD, median (interquartile range) or n (%). CAD, coronary artery disease; LAD, left anterior descending artery; LCX, left circumflex; LM, left main; RCA, right coronary artery; PCI, percutaneous coronary intervention; SS, SYNTAX score; SS II, SYNTAX score II.

Supplemental Table 2. Five-Years cumulative incidence of adverse events

|  | Low SS II  (a) | Median SS II  (b) | High SS II  (c) | P value | | | |
| --- | --- | --- | --- | --- | --- | --- | --- |
|  |  |  |  | Trend | a vs. b* | a vs. c* | b vs. c* |
| All-cause mortality | 3.0% (44) | 9.2% (70) | 16.0% (40) | <0.001 | <0.001 | <0.001 | 0.003 |
| Cardiac mortality | 1.5% (22) | 5.3% (41) | 13.5% (34) | <0.001 | <0.001 | <0.001 | <0.001 |
| Myocardial infarction | 5.1% (74) | 4.4% (34) | 13.0% (32) | 0.005 | 0.613 | 0.002 | 0.014 |
| Unplanned  revascularization | 11.9% (173) | 12.5% (96) | 9.5% (24) | 0.769 | 0.497 | 0.917 | 0.628 |
| Stroke | 7.5% (109) | 6.8% (52) | 14.3% (36) | 0.257 | 0.796 | 0.101 | 0.178 |
| MACCEs | 20.5% (299) | 26.9% (21) | 35.2% (87) | <0.001 | 0.004 | <0.001 | 0.031 |

Event rates are Kaplan-Meier estimates, % (n). *Adjusted significance level is 0.017. MACCEs, major adverse cardiovascular and cerebrovascular events; SS II, SYNTAX score II.
